# Comparison of Maize Genotypes Using Drought-Tolerance Indices and Graphical Analysis under Normal and Humidity Stress Conditions

**DOI:** 10.3390/plants11070942

**Published:** 2022-03-30

**Authors:** Seyed Habib Shojaei, Khodadad Mostafavi, Ali Omrani, Árpád Illés, Csaba Bojtor, Saeed Omrani, Seyed Mohammad Nasir Mousavi, János Nagy

**Affiliations:** 1 Department of Biotechnology and Plant Breeding, Science and Research Branch, Islamic Azad University, Tehran 1477893855, Iran; shs2784@gmail.com; 2 Department of Agronomy and Plant Breeding, Karaj Branch, Islamic Azad University, Karaj 3149968111, Iran; mostafavi@kiau.ac.ir; 3 Crop and Horticultural Science Research Department, Ardabil Agricultural and Natural Resources Research and Education Center, AREEO, Moghan 193951113, Iran; a.omrani@areeo.ac.ir; 4 Institute of Land Use, Engineering and Precision Farming Technology, Faculty of Agricultural and Food Sciences and Environmental Management, University of Debrecen, 138 Böszörményi St., 4032 Debrecen, Hungary; illes.arpad@agr.unideb.hu (Á.I.); bojtor.csaba@agr.unideb.hu (C.B.); nagyjanos@agr.unideb.hu (J.N.); 5 Plant Breeding and Genetics, Department of Agronomy and Plant Breeding, Isfahan University of Technology, Isfahan 84156-83111, Iran; s.omrani70@gmail.com

**Keywords:** maize, drought-tolerance, correlation, graphic analysis, drought stress

## Abstract

This study aimed to identify drought-tolerant genotypes and to evaluate and compare the response of genotypes under normal conditions and humidity stress. The experiment was conducted in a Randomized Complete Block Design (RCBD) on 12 commercial single cross hybrids of maize (*Zea mays* L.) with three replications in two separate experiments under normal and stress conditions. GT biplot was used to compare genotypes under normal conditions and humidity stress. Based on the polygon diagrams’ graphical analysis, KSC206, KSC704, KSC705 and KSC706 genotypes were identified as desirable hybrids. The ranking diagram of genotypes based on ideal genotype also showed that the KSC704 genotype had high desirability in all evaluated traits in normal and stress conditions. TOL, MP, HARM, GMP, SSI and STI indices were used to identify drought-tolerant genotypes, and the genotypes were ranked based on this index. Based on this, KSC260, SC302 and KSC400 hybrids were selected as resistant hybrids. Based on the correlation analysis between drought-tolerance indices, a positive correlation was observed between MP, GMP, HARM and STI indices. Based on the analysis of the PCA on the indices, the first and second principal components were given the titles of grain yield tolerance component under humidity stress conditions and grain yield stability component under normal humidity conditions, respectively. KSC704 was superior to other hybrids in terms of grain yield under normal conditions and stress, and the KSC260 hybrid was identified as a tolerant hybrid in terms of all studied traits under drought stress.

## 1. Introduction

Corn (*Zea mays* L.) is an annual monocotyledonous plant of the cereal family [1]. After wheat and rice, it is the third most important crop among cereals [2]. Environmental stress is one of the most important factors in reducing the yield and production of crops. To increase the yield of these products, dealing with the effects of stress is considered one of the useful methods [3]. Among abiotic stresses, drought stress is one of the biggest environmental constraints that reduces and limits crop production [4]. Drought stress is one of the most important constraints on agricultural production in most developing countries located in arid and semi-arid regions of the world. Drought stress is one of the most important factors limiting yield in maize [5]. One of the first needs of corn cultivation is water required for irrigation, which is declining in most parts of the world [6]. The most common drought-tolerance indices are the tolerance index (TOL) [7], harmonic mean (HARM) [8], mean productivity (MP), geometric mean productivity (GMP), and stress-tolerance index (STI) [9], and stress-sensitivity index (SSI) [10] to identify drought-tolerant hybrid. In most experiments, the correlation between TOL and SSI indices is positive, and selection based on SSI index is effective in low-yield genotypes under stress conditions and in high-yield genotypes under stress conditions [11]. Based on Ranjbar and Rousta’s study on wheat genotypes, the STI index was introduced as an effective indicator in genotype selection [12]. In another study conducted by Sedri, it was reported as the best indicator for selecting maize hybrids in STI stress conditions [13]. The GGE biplot method can be used to analyze multivariate experiments; this method can also be used in line × tester, genotype × environment and genotype × traits [14,15]. The GGE biplot method, due to its high flexibility in analysis, can graphically play a very important role in selecting the desired genotypes [14]. To investigate the interaction between genotype and trait (GT biplot), one of the GGE biplot methods, Yan and Rajkan used different traits in the studied genotypes in their experiments [16]. A study of eight commercial maize hybrids used drought-tolerance indices to select the most tolerant genotypes under stress conditions [17]. Many studies have been conducted on various plants using drought-tolerance indices, including wheat [18] and safflower [19]. Drought-tolerance depends on morphological and physiological characteristics in plants. Morphological traits correlated with drought-tolerance included early maturity; shape; the size and structure of stomata; size; the number and direction of leaves; the presence of cuticle, waxiness of stem, or leaf blade and rooting pattern; and physiological traits including photosynthesis rate, transpiration rate, osmotic concentration, etc., which are different genotypes due to the poly-genetic nature of these traits [20]. One of the most important factors in selecting particle hybrids (*Zea mays* L.) is phenotypic evaluation, and high yield under normal conditions and humidity stress in a specific area. This study investigated the relationship between different traits with grain yield, selected the most important morphological characteristics affecting grain yield under normal conditions and humidity stress, and determined the most tolerant hybrid under stress conditions using drought-tolerance indices. The purpose of this study includes: (1) to compare traits in maize hybrids under normal conditions and water stress, (2) to study and select stress-resistant genotypes using drought-tolerance indices, (3) to investigate the correlation between traits under normal and stress conditions, (4) selection of superior genotypes based on the evaluated traits, and (5) investigation of the relationship between grain yield traits and traits related to grain yield.

## 2. Results and Discussion

### 2.1. Analysis of Variance and Mean Comparison

Analysis of variance in terms of traits was performed on the tested hybrids. Under normal humidity conditions, different hybrids had significant differences in all traits except plant height, number of rows per ear, grain length and grain thickness. Under stress conditions, genotypes showed significant differences in all traits except plant height, the number of grains per row, grain width and grain thickness. In both normal and stress conditions, the highest percentage of coefficient of variation was related to the grain thickness trait, and the lowest was related to the ear length trait (*p* ≤ 0.01) (Table 1). The significant difference between different genotypes in maize yield and yield-dependent traits indicates genetic diversity and the possibility of selection for genotype tolerant to drought stress [21]. Comparing the mean of Duncan method genotypes under normal and stress conditions showed that KSC707, SC301 and KSC704 genotypes had better performance than other hybrids in all evaluated traits, respectively. Additionally, the DC307 genotype under normal conditions and the KSC400 genotype under stress conditions were identified as hybrids with low yield and rank (Table 2), Mousavi et al. [22]. In their experiments under normal humidity conditions, the KSC704 genotype had the highest yield compared to other hybrids.

### 2.2. Analysis of Correlations between Traits

**Figure 1:**
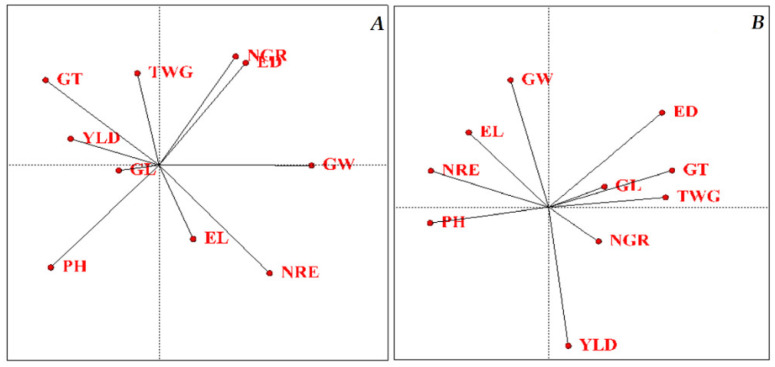
Correlation diagram between the studied traits under normal conditions and water stress. (**A**): normal conditions; (**B**): stress conditions. PH: Plant height, EL: Ear length, ED: Ear diameter, NGR: Number of grains in a row, NRE: Number of rows in-ear, GW: Grain weight, GL: Grain Length, GT: Grain Thickness, TWG: Thousand-grain weight, and YLD: Grain yield.

The correlation coefficient matrix for normal humidity conditions also showed the number of grains per row with the trait of ear width, the number of rows per ear with the number of grains per ear, the grain width trait with the ear width, and the grain length trait with the ear length; the trait Grain thickness had a positive and significant correlation with grain width and grain yield with grain length and 1000-grain weight. There was also a significant negative correlation between ear width, number of grains per row and grain width–plant height, grain thickness-ear length, grain yield-ear width, grain length and grain thickness-number of rows per ear. The results of correlation coefficients under stress conditions also showed a positive and significant correlation between ear length, grain length and 1000-grain weight-plant height, ear width, number of seeds per row and number of rows per ear-ear length, grain width, grain thickness, 1000-grain weight and grain yield-ear width, grain thickness-number of rows per ear, grain length and grain yield-grain thickness, 1000-grain weight-grain length and grain thickness, and grain yield-1000-grain weight. Additionally, a negative and significant correlation was observed between the number of rows per ear with the ear’s width, the grain thickness with the grain length and the trait of 1000-grain weight with the grain width (Table 3). Refiq et al. The authors of [23] reported a significant positive correlation between 1000-grain weight and grain yield in the plot. To investigate the correlation of the studied traits, a graphical analysis of the correlation between the traits was used (Figure 1). In this cosine biplot diagram, the angle between the trait vectors indicates the intensity of the correlation between the traits. Suppose the angle between the vectors is less than 90 degrees. In that case, the correlation between the vectors is equal to +1. If the angle between the vectors of the attributes is 90 degrees, the correlation between the vectors of the attributes is zero. If the angle between the vectors is 180 degrees, the correlation is −1 [24]. Based on the graph obtained under normal conditions (Figure 1A); the number of grains per row and ear width together; the number of rows per ear and ear length together; the plant height traits with grain length; and finally, the grain yield traits, grain length, grain thickness and 1000-grain weight showed a positive and significant correlation. The 180-degree angle between the plant height and ear width vectors showed a significant negative correlation between these two traits. Based on the graph obtained under stress conditions (Figure 1B), ear width; grain thickness; ear length; 1000-grain weight together and grain width; ear length and number of rows per ear together; and, finally, grain yield trait with the number of grain per row had a positive correlation. A negative correlation was observed between plant height and 1000-grain weight and grain yield with grain width, Farajzadeh et al. [25]. In the study of grain yield and yield components of 22 maize genotypes, a positive and significant correlation was observed in the number of grains per row, the number of grains per ear, and ear length with grain yield [21]. Mousavi et al. also reported a significant positive correlation between grain yield traits and many grains per row [26].

### 2.3. Ranking and Grouping of Genotypes in Terms of Traits

**Figure 2:**
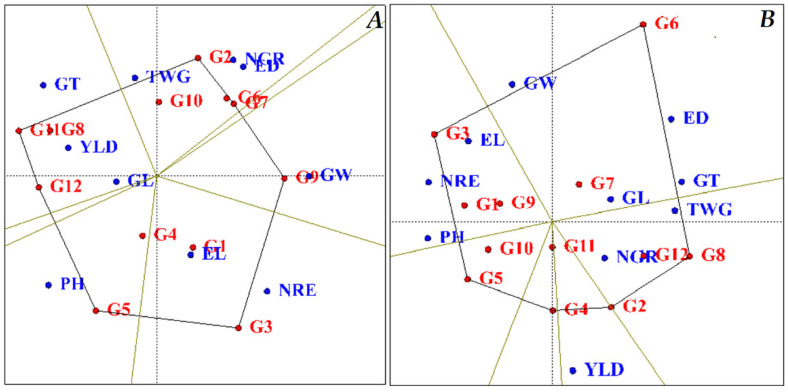
Ranking and Grouping of Genotypes in Terms of Traits. (**A**): normal conditions; (**B**): stress conditions. PH: Plant height, EL: Ear length, ED: Ear diameter, NGR: Number of grains in a row, NRE: Number of rows in-ear, GW: Grain weight, GL: Grain Length, GT: Grain Thickness, TWG: Thousand-grain weight, and YLD: Grain yield. G1: KSC703, G2: KSC260, G3: KSC705, G4: KSC400, G5: KSC706, G6: KSC704, G7: KSC707, G8: DC370, G9: SC647, G10: SC302, G11: SC604, and G12: SC301.

A polygon diagram identifies the best genotypes among the studied traits. This diagram is drawn by connecting the genotypes farthest from the origin so that the other genotypes fit into this polygon. In each section, genotypes with higher yield and desirability with specific traits are separated by lines [27,28]. The authors of [29] used this type of graph for their studies on rapeseed cultivars and maize cultivars [29]. Based on the polygon diagram obtained under normal humidity conditions (Figure 2A), KSC260, KSC704, KSC707, SC647, KSC705, KSC706, SC301 and SC604 hybrids had the longest distance from the origin of the diagram. They were placed at the vertex of the polygon. Titles of desirable hybrids were identified in terms of traits. In each section, KSC260 hybrid in terms of the number of grains per row and ear width, SC647 hybrid in terms of grain width, KSC705 hybrid in terms of a number of rows per ear, KSC706 genotype in terms of plant height, and SC604 and DC370 genotypes in terms of traits grain yield and grain thickness were identified as more favorable hybrids than other genotypes (Figure 2A). The diagram obtained under stress identified KSC704, DC370, KSC260, KSC400, KSC706 and KSC705 genotypes as more favorable genotypes than other genotypes. In each section, DC370 and SC301 genotypes were identified in terms of numbers of grain per row and KSC705 hybrid in terms of ear length and number of rows per ear as high-performance genotypes in these traits (Figure 2B). Considering the comparison of normal and stress conditions, it can be concluded that based on this diagram, KSC260, KSC704, KSC705 and KSC706 genotypes are identified as desirable hybrids in both conditions. In terms of adjective, the number of rows per ear shows good stability and performance.

### 2.4. Ranking of Genotypes Based on Ideal Genotype

**Figure 3:**
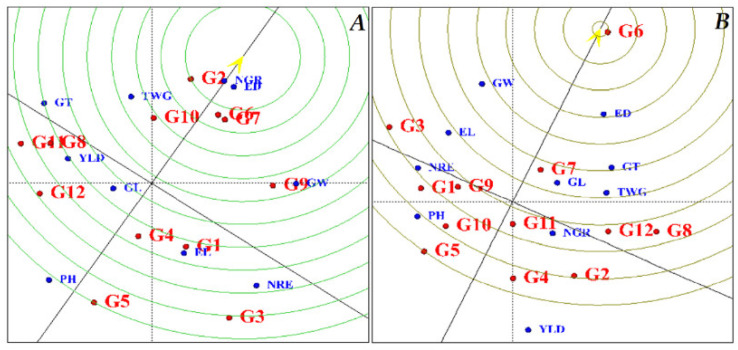
Diagram of the reaction of maize hybrids based on the ideal genotype under normal conditions and water stress. (**A**): normal conditions, (**B**): stress conditions. PH: Plant height, EL: Ear length, ED: Ear diameter, NGR: Number of grains in a row, NRE: Number of rows in-ear, GW: Grain weight, GL: Grain Length, GT: Grain Thickness, TWG: Thousand-grain weight, and YLD: Grain yield. G1: KSC703, G2: KSC260, G3: KSC705, G4: KSC400, G5: KSC706, G6: KSC704, G7: KSC707, G8: DC370, G9: SC647, G10: SC302, G11: SC604, and G12: SC301.

According to the genotype-ranking diagram, the ideal genotype (Figure 3) is connected to the mean point from the origin of the coordinates of the linear graph and continues to both sides. In this form, the best point is the center of the concentric circle, which is marked with an arrow, and other genotypes are ranked according to this point. Based on the diagram obtained under normal moisture conditions (Figure 3A), KSC260 and KSC704 genotypes were preferred to other hybrids. KSC706 and KSC705 genotypes were also identified as unfavorable genotypes. The order of genotypes from the best hybrid to the most unfavorable hybrid is as follows:

KSC260 > KSC704 > KSC707 > SC302 > SC647 > DC370 > SC604 > SC301 > KSC400 > KSC703 > KSC705 > KSC706.

In the diagram obtained under stress conditions, KSC704 and KSC707 hybrids were identified as desirable hybrids and KDC260, KSC400 and KSC706 genotypes were based on the ideal genotype unfavorable hybrids (Figure 3B). The order of genotypes from the best genotype to the most unfavorable genotype in stress conditions is as follows:

KSC704 > KSC707 > KSC705 > SC647 > SC604 > SC301 > DC370 > KSC703 > SC302 > KSC260 > KSC400 > KSC706.

### 2.5. Grouping of Hybrids

**Figure 4:**
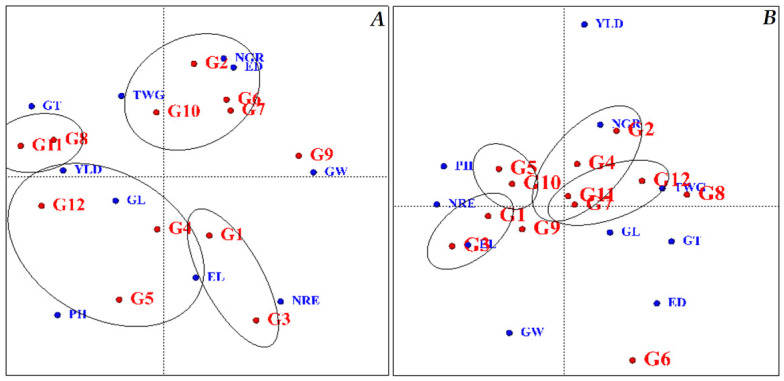
Graphing diagram of Maize hybrids based on the traits studied in the experiment under normal conditions and moisture stress. (**A**): normal conditions, (**B**): stress conditions. PH: Plant height, EL: Ear length, ED: Ear diameter, NGR: Number of grains in a row, NRE: Number of rows in-ear, GW: Grain weight, GL: Grain Length, GT: Grain Thick-ness, TWG: Thousand-grain weight, and YLD: Grain yield. G1: KSC703, G2: KSC260, G3: KSC705, G4: KSC400, G5: KSC706, G6: KSC704, G7: KSC707, G8: DC370, G9: SC647, G10: SC302, G11: SC604, and G12: SC301.

The genotype grouping diagram evaluates hybrids based on stability and yield in different traits and groups the genotypes based on the traits (Figure 4). Based on the grouping diagram under normal humidity conditions, four groups were formed regarding yield and desirability in all traits. The first group included KSC260, KSC704, KSC707 and SC302 genotypes; the second group included DC370 and SC604 genotypes; the third group included SC301 KSC400 KSC706 genotypes; and the fourth group included KSC703 and KSC705 genotypes. The SC647 genotype was not grouped (Figure 4A). Under stress conditions, the grouping diagram classified the genotypes into four groups. The first group included KSC707, SC301 and SC604 genotypes; the second group included KSC260, KSC400, SC604 and KSC707 genotypes. In these two groups, two hybrids, KSC707 and SC604, were common between these groups. KSC706 and SC302 genotypes were in the third group, and KSC703 and KSC705 were in the fourth group. In this diagram, DC370, SC647 and KSC704 genotypes were not in any group (Figure 4B). By examining the graphs of normal and stress conditions, KSC703 and KSC705 genotypes were in the same group in both conditions, indicating the stability of these two genotypes in terms of the studied traits under stress.

### 2.6. The Centred Scatter Plot

**Figure 5:**
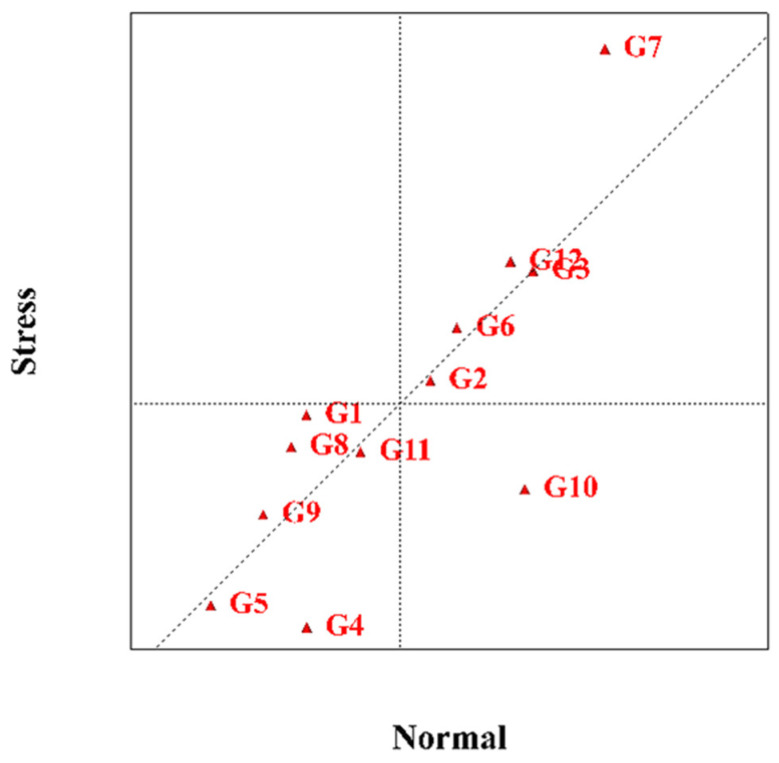
The Centred Scatter Plot Maize hybrids examined in experiments under normal conditions and humidity stress. G1: KSC703, G2: KSC260, G3: KSC705, G4: KSC400, G5: KSC706, G6: KSC704, G7: KSC707, G8: DC370, G9: SC647, G10: SC302, G11: SC604, and G12: SC301.

This diagram is a two-dimensional graph used to compare genotypes in two different positions or compare different positions and test environments in two genotypes. This diagonal linear diagram is divided into two parts and shows compatible and stable genotypes in each environment. According to Figure 5, which shows the genotypes in terms of all the traits evaluated in the experiment under normal conditions and moisture stress, KSC400 and SC302 hybrids are among the hybrids that have good performance in all traits under normal conditions. KSC707, KSC703 and DC370 are also hybrids that have better performance under drought stress conditions. The rest of the genotypes were identified as stable intermediate hybrids in both conditions due to their proximity to the line separating the normal and stress positions.

### 2.7. Evaluation of Drought Stress Using Drought-Tolerance Indices

Drought-tolerance indices were analyzed to evaluate the evaluated hybrids under drought stress conditions (Table 4). The highest and lowest mean yields under normal conditions of humidity and stress did not belong to a specific genotype, so the use of stress tolerance and sensitivity indices is effective in evaluating genotypes. According to the Drought-Tolerance Index (TOL), which is obtained from the difference in the performance of each genotype under normal and stress conditions, tolerant hybrids are considered to be less than this index [30]. Based on this index, the KSC260 genotype was the most resistant hybrid with 1.38, and in the second and third ranks were DC370 (1.42) and KSC400 (1.53) hybrids, respectively. The highest TOL index was related to the SC302 genotype (2.34). Based on the mean productivity index (MP), genotypes are tolerated that have a higher value of this index [7]. Based on this index, KSC260 (6.95), SC302 (6.83) and KSC400 (6.63) genotypes as tolerant genotypes and KSC704 (5.245) and SC647 (5.635) hybrids as sensitive hybrids were identified. Based on the Harmonic Mean (HARM), the genotype with the highest index value was identified as the resistant genotype. Based on this, KSC260 (6.881), SC302 (629.6) and KSC400 (6.546) hybrids were identified as resistant hybrids, and KSC704 (0.512) and SC647 (5.525) genotypes were identified as susceptible hybrids. Based on the Geometric Mean Performance Index (GMP), tolerant genotypes accounted for more of this index. Accordingly, KDC260 (6.91), SC302 (6.72) and KSC400 (6.59) hybrids were identified as resistant hybrids, and KSC704 (5.12) and SC647 (5.58) genotypes were identified as susceptible hybrids. According to the Stress Sensitivity Index (SSI), which is mostly used to remove sensitive genotypes, any genotype with higher values of this index is more sensitive to stress [10]. Accordingly, hybrids of KDC260 (0.71), DC370 (0.81) and KSC400 (0.82) as the most resistant hybrids and genotypes SC302 (1.6) and KSC704 (1.38) as susceptible genotypes were identified. According to the stress tolerance index (STI), the higher the value of this index, the more tolerance of the genotype, based on the genotypes KSC260 (17.3), SC302 (16) and KSC400 (15.9) as resistant genotypes, and KSC704 (10.4) and SC647 (12.2) hybrids were identified as susceptible hybrids. Based on the results obtained from Table 4, it can be concluded that based on drought-tolerance indices on hybrids studied in this experiment, KSC260, SC302 and KSC400 hybrids are drought-tolerant hybrids. KSC704 and SC647 genotypes were identified as susceptible hybrids (Table 4). Table 5 also shows the selected hybrids based on drought-tolerance indices.

### 2.8. Correlation of Drought-Tolerance Indices

**Figure 6:**
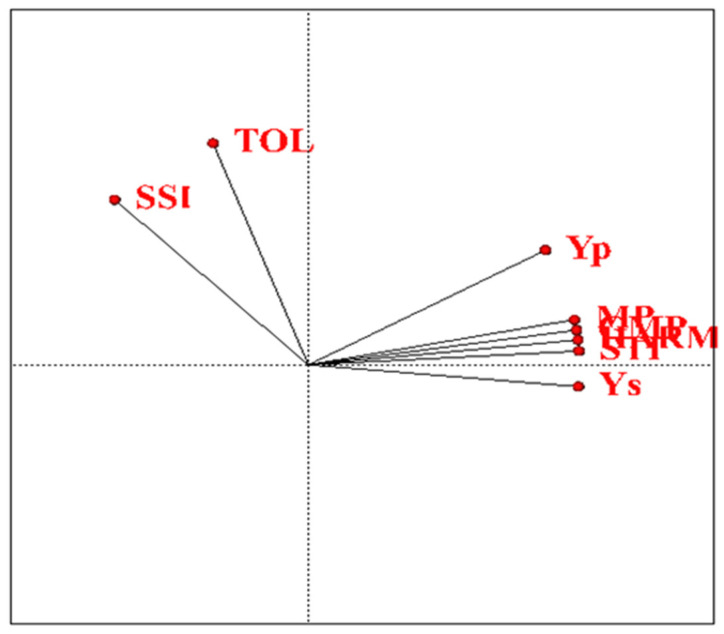
Correlation diagram between drought-tolerance indices studied under water stress conditions. Yp: Yield under normal condition, Ys: yield under drought condition, TOL: Tolerance, MP: Mean Productivity, GMP: Geometric Mean Productivity, HARM: Harmonic Mean Productivity, SSI: Stress Susceptibility Index, STI: Stress Tolerance Index.

Correlation coefficients based on data obtained from grain yield under normal humidity and stress conditions with drought-tolerant indices showed that TOL, MP, HARM and SSI indices with average grain yield under normal humidity conditions (Yp); index GMP with mean grain yield under stress (Ys); MP, HARM and SSI indices with TOL index; HARM and SSI indices with MP index; and SSI index with HARM index had a positive and significant correlation at the probability level of 0.01. (Table 6). Additionally, based on the correlation diagram drawn between the data obtained from the average grain yield under normal conditions of moisture (Yp) and moisture stress (Ys) as well as drought-tolerance indices, it can be concluded that between MP GMP, there is a significant positive correlation between HARM, STI, Yp and Ys. According to the 90-degree angle between the vectors of MP and TOL, the correlation was estimated to be zero (Figure 6). Many researchers have reported a significant positive correlation between Yp and Ys, suggesting that high-yielding genotypes under normal conditions can perform well under stress conditions [13,30].

### 2.9. Polygon Diagram

**Figure 7:**
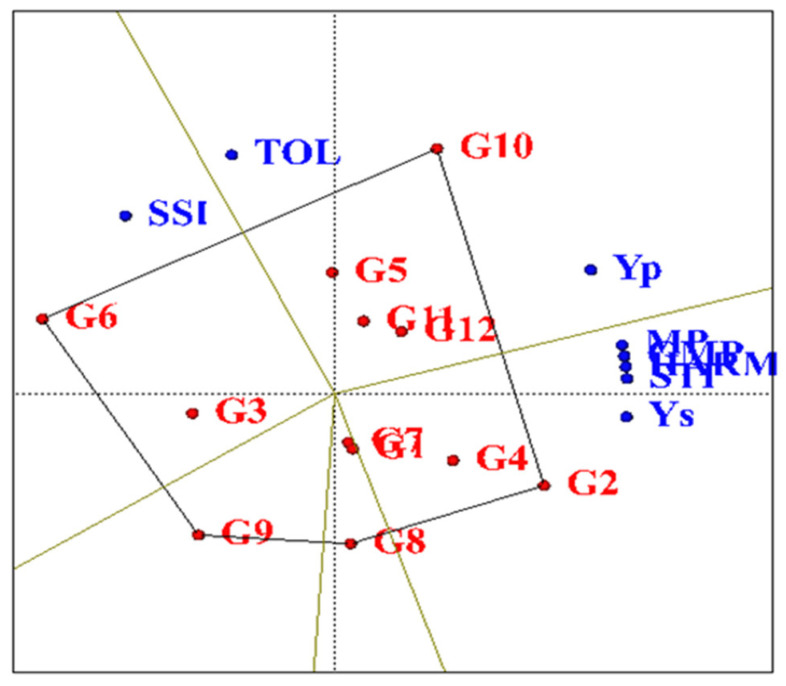
Diagram of a polygon diagram of the reaction of maize hybrids to drought-tolerance indices studied under water stress conditions. Yp: Yield under normal condition, Ys: yield under drought condition, TOL: Tolerance, MP: Mean Productivity, GMP: Geometric Mean Productivity, HARM: Harmonic Mean Productivity, SSI: Stress Susceptibility Index, STI: Stress Tolerance Index. G1: KSC703, G2: KSC260, G3: KSC705, G4: KSC400, G5: KSC706, G6: KSC704, G7: KSC707, G8: DC370, G9: SC647, G10: SC302, G11: SC604, and G12: SC301.

Based on the obtained polygon diagram in terms of drought-tolerance indices (Figure 7), SC302, KSC260, DC370, SC647 and KSC704 genotypes were identified as more favorable hybrids than other evaluated hybrids. Additionally, in each section, the KSC260 genotype was more desirable than other genotypes in MP, GMP, STI, HARM and Ys indices. The KSC704 genotype was superior to other genotypes in the SSI index. In his study on wheat genotypes, Karaman used this type of graph to investigate the response of different genotypes to drought-tolerance indices [31].

### 2.10. Principal Components Analysis in Drought-Tolerance Indices

After analyzing drought-tolerance indices and mean grain yield under normal conditions and moisture stress in the studied hybrids, based on principal component analysis, the most changes were expressed in the first two components, and more than 99% of the data variance by the two components was justified (Table 7). The first component accounted for more than 78% of the data variance in this analysis. This component showed a high correlation with the average performance under water stress (Ys), MP, HARM, GMP and STI indices. A negative correlation was identified with TOL and SSI indices. Hence, under stress conditions, the first component was named the grain yield tolerance component. The second component explained more than 20% of the data variance. A positive correlation was observed with the mean grain yield under normal conditions (Yp), and the highest correlation was with TOL and SSI indices. This component negatively correlated with the average grain yield under moisture stress (Ys) and was named the grain yield stability component under normal moisture conditions. In their study, Ali and El-Sadek evaluated drought-tolerance indices using the analysis of principal components under stress and non-stress conditions. As a result, the first two components comprised more than 98% of the total changes related to the index for drought tolerance [32].

## 3. Materials and Methods

In this experiment, the effect of drought stress on grain yield and morphological characteristics and yield components, as well as a comparison of 12 commercial single cross hybrids (Table 8) under normal conditions and humidity stress in a randomized complete block design (RCBD) in three replications in the research field Islamic Azad University, Karaj Branch, was examined. Karaj region has a longitude of ‘54°50′ E’ and latitude of ‘55°35′ N’, is 1312 m above sea level and has an average annual rainfall of 247.3 mm. A separate experiment was considered for each environmental condition (normal and drought stress). Specifications of each experimental plot were planted, including four lines with a length of 2 m and planting lines with a distance of 75 cm. Planting, holding and harvesting operations were performed accurately under normal conditions and humidity stress. It was determined based on soil sampling and 50% (normal irrigation), and stress was applied to apply irrigation stress. Sampling and taking notes were performed from the two middle rows and the plant height pre-harvest and other post-harvest traits. The studied traits include plant height (PH), ear length (EL), ear diameter (ED), number of seeds per row (NGR), number of rows per ear (NRE), grain width (GW), grain length (GL), grain thickness (GT), 1000-grain weight (TWG) and grain yield (YLD). (Table 8). The soil characteristics of the cultivated area are presented in Table 9.

To calculate drought-tolerance indices from tolerance index (TOL), mean productivity (MP), harmonic mean (HARM), geometric mean productivity (GMP), stress sensitivity index (SSI) and stress tolerance index (STI), the following formulas were used:

(1) TOL=Yp−Ys

(2) $$\mathrm{MP}=\frac{\mathrm{Yp}+\mathrm{Ys}}{2}$$

(3) $$\mathrm{HARM}=\frac{2\left(\mathrm{Yp}\times \mathrm{Ys}\right)}{\mathrm{Yp}+\mathrm{Ys}}$$

(4) $$\mathrm{GMP}=\sqrt{\left(\mathrm{Yp}\right)\left(\mathrm{Ys}\right)}$$

(5) $$\mathrm{SSI}=\frac{\left(\frac{\mathrm{Ys}}{\mathrm{Yp}}\right)}{1-\left(\frac{Ӯs}{Ӯp}\right)}$$

(6) $$\mathrm{STI}=\frac{\mathrm{Yp}\times \mathrm{Ys}}{\mathrm{Yp}-\mathrm{Ys}}$$

In these equations, Yp is the average yield under normal moisture conditions, Ӯp is the average yield of all genotypes under normal moisture conditions, Ys is the average yield under moisture stress conditions and Ӯp is the average yield all genotypes under drought stress conditions.

For studying the genotype × trait interaction, Yan and Rajcan [16] method was used as below (Equation (7)):

(7) $$\frac{\alpha_{\mathrm{ij}}-\beta_{j}}{\sigma_{j}}=\overset{2}{\underset{n=1}{\sum }}\lambda_{n}\xi_{\mathrm{in}}\eta_{\mathrm{jn}}+\varepsilon_{\mathrm{ij}}=\overset{2}{\underset{n=1}{\sum }}\xi_{\mathrm{in}}^{*}\eta_{\mathrm{jn}}^{*}+\varepsilon_{\mathrm{ij}}$$

where α_ij_: the average amount of genotype i for every trait j, β_j_: the average amount of all the genotypes for the traits, and σ_j_: standard deviation of the trait j in the average genotypes. ε_ij_: the amount of genotype i remained in the trait j, λ_n_: certain amount for the main element (PC_n_), ξ_i_: the amount of PC_n_ for the genotype i, and η_jn_: the amount of PC_n_ for the genotype j.

SAS.v9.2 software was used in the statistical analyzes, which included analysis of variance, comparison of means by Duncan method, correlation coefficients between traits and drought-tolerance indices, and principal components analysis (PCA). Excel software was also used to analyze drought-tolerance indices, and Genstat.v12 software was used to analyze correlation graphically, polygon diagrams, rank genotypes based on ideal genotype, the grouping of genotypes, and Centered Scatter Plot.

## 4. Conclusions

KSC260, SC302 and KSC400 hybrids were identified as drought-tolerant hybrids, and KSC704 and SC647 genotypes were identified as susceptible hybrids based on drought-tolerance indices for the hybrids studied in this experiment. KSC260, KSC704, KSC705 and KSC706 genotypes are identified as desirable hybrids in both conditions. It can be concluded based on this diagram that the number of rows per ear shows good stability and performance in terms of adjectives. Based on the correlation coefficients of drought-tolerance indices, mean grain yield under normal moisture conditions (Yp) with TOL, MP, HARM and SSI indices and mean grain yield under humidity stress (Ys) with GMP index had a positive and significant correlation. The principal components (PCA) analysis on drought-tolerance indices also showed that the first two components explained more than 99% of the variance. The first component was the grain yield tolerance component under stress conditions, and the second component was the grain yield stability component under conditions. Finally, it can be concluded that the KSC704 hybrid as a hybrid was superior to other studied hybrids in terms of grain yield under normal conditions and stress and the KSC260 hybrid was superior as a hybrid in terms of all studied traits in drought stress.

## Figures and Tables

**Table 1:** Analysis of variance of studied hybrids in terms of traits under normal conditions and humidity stress.

		MS										
State	S.O.V.	DF	PH	EL	ED	NGR	NRE	GW	GL	GT	TWG	YLD
Normal Condition	Block	2	110.1 ^ns^	0.06 *	9.59 *	4.68 *	31.6 ^ns^	0.57 *	1.31 ^ns^	1.27 ^ns^	277.6 ^ns^	0.21 ^ns^
	Genotype	11	519.2 ^ns^	10.8 **	62.6 **	9.48 *	73.79 ^ns^	2.91 **	5.95 ^ns^	2.21 ^ns^	3961.9 *	0.77 *
	Error	22	331.9	1.69	14.47	3.56	53.3	0.75	4.8	1.87	3035.2	1.83
	CV%	---	9.91	7.8	9.28	11.46	19.3	15.81	22.8	30.16	18.9	18.87
Stress Condition	Block	2	577.6 ^ns^	0.36 ^ns^	10.08 ^ns^	1.33 ^ns^	17.33 ^ns^	0.13 ^ns^	8.14 *	8.37 ^ns^	6385.08 *	2.33 *
	Genotype	11	390.35 ^ns^	8.96 **	40.5 *	6.5 ^ns^	68.2 *	0.66 ^ns^	4.5 *	1.45 ^ns^	2037.8 *	0.91 *
	Error	22	832.3	2.55	19.93	3.87	35.39	0.73	2.63	1.29	2083.3	1.23
	CV%	---	22.5	13.19	17.38	15.54	21.5	23.03	22.4	28.8	23.9	20.72

*, **, and ns: significant at 5%, 1% and not-significant. PH: Plant height, EL: Ear length, ED: Ear diameter, NGR: Number of grains in a row, NRE: Number of rows in-ear, GW: Grain weight, GL: Grain Length, GT: Grain Thickness, TWG: Thousand-grain weight, and YLD: Grain yield.

**Table 2:** Comparison of Duncan’s mean for traits in 12 hybrids of maize under normal conditions and humidity stress.

	Genotype	Rank	PH	EL	ED	NGR	NRE	GW	GL	GT	TWG	YLD
Normal Condition	KSC703	7	182ab	17.7b	38.4cde	15.1bc	39.1ab	6.3ab	9.4ab	2.9bc	258.4ab	7.1abc
	KSC260	5	162.4b	17.1bc	42.3abcd	19.7a	35.6ab	5.5abc	6.8b	4.1ab	316.6ab	7.64ab
	KSC705	6	197.8ab	21.4a	40.9bcd	15.1bc	48.6a	5.9ab	8.4ab	2.7bc	290.5ab	6.65bc
	KSC400	10	185.9ab	14.8cd	36.8de	14.5c	40.2ab	5.6ab	10.9ab	2.6bc	297.1ab	7.4ab
	KSC706	9	198.7a	17.1bc	32.5e	14.6c	40.8ab	4.9bcd	8.6ab	3.7abc	225.8ab	7.4ab
	KSC704	3	167.3ab	16.8bcd	49.4a	16.7abc	37.7ab	5.8ab	10.2ab	4.3ab	297.8ab	6.35bcd
	KSC707	1	171.9ab	16.8bcd	42.9abcd	18.4ab	38.1ab	7.01a	10ab	3.6abc	336.6a	7.1abc
	DC370	11	160.8ab	14.4d	38.4cde	14.5c	30.5b	4.9bcd	10.2ab	4.6ab	337.4a	6.9bc
	SC647	8	176.6ab	14.4d	44.3abc	18.7ab	40.5ab	6.5ab	9.8ab	2.4c	232.6ab	6.42bcd
	SC302	4	176.2ab	15.6bcd	46.2ab	17abc	36.3ab	5.5abc	9.17ab	3.5abc	310.5ab	8a
	SC604	12	202.7a	16.1bcd	41.8abc	16.9abc	29.9b	3.6d	8.5ab	5.1a	276.6ab	7.4a
	SC301	2	193.1ab	17.6b	37.5cde	15.9bc	35.2ab	3.9cd	12.4a	4.2ab	306ab	7.5ab
Stress Condition	KSC703	5	137.3abc	13.3ab	22.8bc	12b	28abc	4.26ab	7.2abc	1.76cd	161.6cd	5.45bc
	KSC260	6	110de	11.26bcd	27.1abc	16a	27.3abc	3.4bc	5.48c	2.28bcd	195bc	6.26a
	KSC705	4	133bcd	15.8a	23.9bc	12.6ab	36.6a	4.13ab	7.2abc	1.3d	188.6bcd	4.78cd
	KSC400	12	121cde	9.3d	23.2bc	12b	26abc	3.4bc	8.9ab	1.48cd	166.6cd	5.87ab
	KSC706	11	136abc	12.6bc	20.4c	11.3bc	32.6ab	3c	6.06bc	2.4bcd	166.3cd	5.29bc
	KSC704	3	108.6e	12.4bc	34.2a	11.3bc	26.6abc	4.5a	7.8abc	3.42a	193.6bc	4.14d
	KSC707	1	134bc	13.13ab	25.5abc	14.6ab	31.3abc	3.93bc	7.03abc	2.68bc	228ab	5.43bc
	DC370	7	119.6de	9.66cd	29.2ab	12.6ab	20c	3.3bc	7.2abc	2.93bc	236a	5.48bc
	SC647	9	141.6a	11.66bcd	25.7bc	13.3ab	25.3abc	4.13ab	7.2abc	1.65cd	155.3d	4.85cd
	SC302	8	133.6bcd	11.96bcd	24.4bc	11.3bc	32ab	3.66bc	5.6c	1.85bcd	188cd	5.66ab
	SC604	10	140ab	12.2bcd	23.2bc	11.3bc	22.6bc	3.5bc	7.05abc	2.95bc	191.3bc	5.44bc
	SC301	2	121.6cd	12.5bc	24.8bc	13.3ab	23.3bc	3.2bc	9.7a	3.09ab	216.6ab	5.6ab

PH: Plant height, EL: Ear length, ED: Ear diameter, NGR: Number of grains in a row, NRE: Number of rows in-ear, GW: Grain weight, GL: Grain Length, GT: Grain Thickness, TWG: Thousand-grain weight, and YLD: Grain yield.

**Table 3:** Correlation coefficients between the evaluated traits under normal conditions and water stress.

Normal Condition		**PH**	**EL**	**ED**	**NGR**	**NRE**	**GW**	**GL**	**GT**	**TWG**
	EL	0.14 ^ns^								
	ED	−0.46 **	−0.08 ^ns^							
	NGR	−0.39 *	−0.1 ^ns^	0.31 *						
	NRE	0.08 ^ns^	0.2 ^ns^	−0.15 ^ns^	0.07 *					
	GW	−0.34 *	−0.1 ^ns^	0.22 *	0.33 ^ns^	0.4 *				
	GL	0.22 ^ns^	0.01 *	−0.14 ^ns^	0.003 ^ns^	−0.04 *	0.03 ^ns^			
	GT	0.28 ^ns^	−0.06 *	0.05 ^ns^	0.04 *	−0.39 *	0.31 *	0.03 ^ns^		
	TWG	0.13 ^ns^	−0.12 ^ns^	0.008 ^ns^	−0.11 *	0.004 ^ns^	0.01 ^ns^	0.1 ^ns^	0.41 *	
	YLD	0.18 ^ns^	0.08 ^ns^	−0.19 *	0.07 ^ns^	−0.003 ^ns^	0.14 ^ns^	0.1 *	0.26 ^ns^	0.46 **
Stress Condition	EL	0.2 *								
	ED	−0.12 ^ns^	0.26 *							
	NGR	−0.14 ^ns^	0.06 *	0.09 ^ns^						
	NRE	0.06 ^ns^	0.51 **	−0.31 *	0.08 ^ns^					
	GW	0.07 ^ns^	0.15 ^ns^	0.03 *	0.19 *	0.23 ^ns^				
	GL	0.53 *	−0.02 ^ns^	−0.16 ^ns^	0.13 ^ns^	−0.06 ^ns^	0.12 *			
	GT	0.02 ^ns^	0.04 ^ns^	0.35 *	0.09 ^ns^	0.29 *	0.14 ^ns^	−0.02 *		
	TWG	0.1 *	0.07 ^ns^	0.01 *	0.05 ^ns^	−0.004 ^ns^	−0.16 *	0.21 *	0.41 *	
	YLD	−0.06 ^ns^	0.04 ^ns^	0.25 *	0.14 ^ns^	0.006 ^ns^	0.1 *	−0.03 ^ns^	0.17 ^ns^	0.29 *

ns: non significant; * *p* < 0.05; ** *p* < 0.01; PH: Plant height, EL: Ear length, ED: Ear diameter, NGR: Number of grains in a row, NRE: Number of rows in-ear, GW: Grain weight, GL: Grain Length, GT: Grain Thickness, TWG: Thousand-grain weight, and YLD: Grain yield.

**Table 4:** Evaluation of hybrids evaluated in the test under stress conditions through drought-tolerance indices.

Genotypes	Yp	R	Ys	R	TOL	R	MP	R	HARM	R	GMP	R	SSI	R	STI	R
KSC703	7.1	8	5.45	6	1.65	5	6.275	7	6.166	7	6.22	7	0.92	4	14.5	6
KSC260	7.64	6	6.26	1	1.38	1	6.95	1	6.881	1	6.91	1	0.71	1	17.3	1
KSC705	6.65	10	4.78	11	1.87	7	5.715	10	5.562	10	5.63	10	1.11	9	12.3	10
KSC400	7.4	5	5.87	2	1.53	3	6.635	3	6.546	3	6.59	3	0.82	3	15.9	3
KSC706	7.4	4	5.29	9	2.11	10	6.345	6	6.169	6	6.25	6	1.13	10	14.3	8
KSC704	6.35	12	4.14	12	2.21	11	5.245	12	5.012	12	5.12	12	1.38	11	10.4	12
KSC707	7.1	7	5.43	8	1.67	6	6.265	8	6.153	8	6.2	8	0.93	5	14.4	7
DC370	6.9	9	5.48	5	1.42	2	6.19	9	6.108	9	6.14	9	0.81	2	14.3	9
SC647	6.42	11	4.85	10	1.57	4	5.635	11	5.525	11	5.58	11	0.97	6	12.2	11
SC302	8	1	5.66	3	2.34	12	6.83	2	6.629	2	6.72	2	1.6	12	16	2
SC604	7.4	3	5.44	7	1.96	9	6.42	5	6.270	5	6.34	5	1.05	8	14.7	5
SC301	7.5	2	5.6	4	1.9	8	6.55	4	6.41	4	6.48	4	1	7	15.3	4

**Table 5:** Selected hybrids based on drought-tolerance indices.

Index	Selected Hybrids
Based on Yp	SC604, SC301, SC302
Based on Ys	SC302, KSC400, KSC260
Based on TOL	KSC400, DC370, KSC260
Based on MP	KSC400, SC302, KSC260
Based on HARM	KSC400, SC302, KSC260
Based on GMP	KSC400, SC302, KSC260
Based on SSI	KSC400, DC370, KSC260
Based on STI	KSC400, SC302, KSC260

**Table 6:** Correlation coefficients between drought-tolerance indices evaluated under water stress conditions.

	Yp	Ys	TOL	MP	Harm	GMP	SSI
Ys	−0.15 ^ns^						
TOL	0.95 **	0.44 ^ns^					
MP	0.97 **	0.36 ^ns^	0.99 **				
Harm	0.96 **	0.4 ^ns^	0.99 **	0.98 **			
GMP	−0.35 ^ns^	0.87 *	−0.05 ^ns^	−0.13 ^ns^	−0.09 ^ns^		
SSI	0.98 **	0.33 ^ns^	0.98 **	0.9 **	0.9 **	−0.18 ^ns^	
STI	−0.36 ^ns^	−0.67 ^ns^	−0.53 ^ns^	−0.49 ^ns^	−0.51 ^ns^	−0.45 ^ns^	−0.46 ^ns^

*, **, and ns: significant at 5%, 1% and not-significant.

**Table 7:** Principal components analysis for average grain yield under normal conditions and moisture stress and drought-tolerance indices.

	% Variance	% Cumulative Variance	Yp	Ys	TOL	MP	Harm	GMP	SSI	STI
Factor 1	0.789	0.789	0.348	0.396	−0.13	0.39	0.395	0.393	−0.28	0.397
Factor 2	0.209	0.999	0.374	−0.07	0.722	0.147	0.081	0.113	0.538	0.045

**Table 8:** Name and code of hybrids, traits and drought-tolerance indices studied in the experiment.

Genotype No.	Genotype	Traits Code	Traits	Indices Code	Indices
G1	KSC 703	PH	Plant height	Yp	Yield under normal condition
G2	KSC 260	EL	Ear length	Ys	Yield under drought condition
G3	KSC 705	ED	Ear diameter	TOL	Tolerance
G4	KSC 400	NGR	Number of grains in row	MP	Mean Productivity
G5	KSC 706	NRE	Number of rows in ear	GMP	Geometric Mean Productivity
G6	KSC 704	GW	Grain weight	HARM	Harmonic Mean Productivity
G7	KSC 707	GL	Grain Length	SSI	Stress Susceptibility Index
G8	DC 370	GT	Grain Thickness	STI	Stress Tolerance Index
G9	SC 647	TWG	Thousand grain weight		
G10	SC 302	YLD	Grain yield		
G11	SC 604				
G12	SC 301				

**Table 9:** Soil characteristics of the cultivated area in the experiment.

Region	EC (ds/m)	Acidity	Lime (%)	Organic Carbon (%)	Organic Materials (%)	Clay (%)	Silt (%)	Sand (%)
Karaj	0.20	8.2	7	32	45	32	25	22

## Data Availability

All data supporting the conclusions of this article are included in this article.
